# A Novel Inactivated Intranasal Respiratory Syncytial Virus Vaccine Promotes Viral Clearance without Th2 Associated Vaccine-Enhanced Disease

**DOI:** 10.1371/journal.pone.0021823

**Published:** 2011-07-15

**Authors:** Dennis M. Lindell, Susan B. Morris, Maria P. White, Lara E. Kallal, Phillip K. Lundy, Tarek Hamouda, James R. Baker, Nicholas W. Lukacs

**Affiliations:** 1 Center for Immunity and Immunotherapies, Seattle Children's Research Institute, Seattle, Washington, United States of America; 2 Department of Pathology, University of Michigan Medical School, Ann Arbor, Michigan, United States of America; 3 NanoBio Corporation, Ann Arbor, Michigan, United States of America; 4 Michigan Nanotechnology Institute for Medicine and Biological Sciences, University of Michigan, Ann Arbor, Michigan, United States of America; University of Liverpool, United Kingdom

## Abstract

**Background:**

Respiratory syncytial virus (RSV) is a leading cause of bronchiolitis and pneumonia in young children worldwide, and no vaccine is currently available. Inactivated RSV vaccines tested in the 1960's led to vaccine-enhanced disease upon viral challenge, which has undermined RSV vaccine development. RSV infection is increasingly being recognized as an important pathogen in the elderly, as well as other individuals with compromised pulmonary immunity. A safe and effective inactivated RSV vaccine would be of tremendous therapeutic benefit to many of these populations.

**Principal Findings:**

In these preclinical studies, a mouse model was utilized to assess the efficacy of a novel, nanoemulsion-adjuvanted, inactivated mucosal RSV vaccine. Our results demonstrate that NE-RSV immunization induced durable, RSV-specific humoral responses, both systemically and in the lungs. Vaccinated mice exhibited increased protection against subsequent live viral challenge, which was associated with an enhanced Th1/Th17 response. In these studies, NE-RSV vaccinated mice displayed no evidence of Th2 mediated immunopotentiation, as has been previously described for other inactivated RSV vaccines.

**Conclusions:**

These studies indicate that nanoemulsion-based inactivated RSV vaccination can augment viral-specific immunity, decrease mucus production and increase viral clearance, without evidence of Th2 immune mediated pathology.

## Introduction

Respiratory syncytial virus (RSV) infection is a major cause of respiratory illness, particularly in infants. It is estimated by the CDC that every year RSV is associated with approximately 125,000 pediatric hospitalizations in the United States, at an annual cost of over $300,000,000 [1]. Despite the generation of RSV-specific adaptive immune responses, RSV infection does not confer protective immunity in humans and recurrent infections are common [2], [3]. While RSV is especially detrimental in very young infants whose airways are small and easily occluded, RSV is also becoming recognized as an important pathogen in transplant recipients, the elderly, and patients with chronic lung diseases, especially chronic obstructive pulmonary disease (COPD) and asthma. Recent data suggest that combined U.S. mortality from RSV in all ages is approximately 30/100,000 from 1990–2000, with an average annual mortality of over 17,000 [4], [5]; however these numbers likely underestimated adult disease, as RSV infection in this populations has not been consistently followed. In infants, severe RSV infection has been linked with the subsequent development of asthma in later childhood. Additionally, RSV is recognized as an important pathogen in the elderly and/or immunocompromised patients, and exacerbation of chronic lung disease. Thus, RSV causes severe lung disease in the young and the elderly. It is particularly a problem in immuno-compromised individuals, and is associated with significant mortality in these populations.

Given the above information, an ideal RSV vaccine would be immunogenic and safe in all the populations susceptible to disease from the infection. In general, however, inactivated RSV vaccines have exhibited poor immunogenicity. Conversely, live-attenuated vaccines, which often cause infection-related symptoms, have had difficulty achieving a balance between immunogenicity and safety. The absence of an effective vaccine is reflected in the mortality rate due to severe RSV. An estimated ninety nine percent of deaths due to RSV occur in the developing world, where supportive therapy is not available [6]. Live attenuated viral vaccines will not solve this problem, as they typically require cold storage prior to use, a significant obstacle to their use in remote areas. Thus, an ambient temperature-stable RSV vaccine would be of significant public health benefit worldwide.

Another unique problem associated with RSV vaccination occurred in the late 1960s, following vaccination of children with an alum-precipitated formalin-inactivated RSV (FI-RSV) vaccine preparation. This vaccine failed, causing severe exacerbated disease upon live RSV infection. Considerable research effort over the past several decades has sought to determine the mechanisms responsible for this phenomenon, referred to as “vaccine-enhanced disease” or “immunopotentiation”. Evidence also clearly demonstrates that the use of alum promotes Th2 responses [7], [8], [9], [10] and therefore the choice of adjuvant must be made carefully.

Nanoemulsion-based mucosal vaccination has been demonstrated to be efficacious in animal models at preventing infection with a number of pathogens, including Anthrax, Influenza, Vaccinia (as a model for smallpox vaccination), HIV, and Hepatitis B [11], [12], [13], [14], [15]. In these studies, we tested the immunogenicity and efficacy of an intranasal nanoemulsion-adjuvanted inactivated RSV vaccine (NE-RSV) in a mouse model of RSV. NE-RSV induced RSV specific IgG and IgA responses, and enhanced viral clearance upon challenge. In contrast to alum, however, it did not result in Th2 mediated immunopotentiation. Our results demonstrate that this novel mucosal vaccine provides a unique approach for effective RSV vaccination.

## Results

### Nanoemulsion effectively inactivates RSV

**Figure 1:**
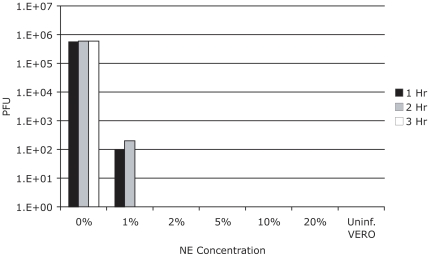
Nanoemulsion effectively inactivates RSV. Nanoemulsion RSV (10^6^ plaque forming units (PFU)) was incubated with nanoemulsion at varying concentrations (0%–20%) and varying times (1 hr–3 hrs). The number of infectious virus particles was determined via plaque assay using Vero cells. Control and nanoemulsion-incubated virus was used to infect sub-confluent Vero cells. RSV plaques at were visualized day 5 post-infection using immunohistochemical techniques.

Previous studies have demonstrated that novel water-in-oil nanoemulsions exhibit broad microbicidal activity [16], [17], [18], [19], [20]. The water-miscible emulsion droplets (<400 nm size) are believed to inactivate viruses by the physical disruption of the viral envelope. To determine whether the nanoemulsion formulation exhibits virucidal activity against RSV, 10^6^ PFU of RSV (Line 19) was incubated with varying concentrations of NE, and then used to infect Type I IFN deficient Vero cells. After the virus was incubated with 2% nanoemulsion for one hour or 1% nanoemulsion for 3 hrs, there was no viable virus as assessed by standard plaque assay (Fig. 1). Inactivation of the virus by NE was further confirmed by serial passage of NE treated RSV in Hep2 cells, followed by plaque assay. After two amplifying passages in Hep2 cells, no plaques were detected from 3 hr incubated NE-RSV, as compared to >10^6^ PFU/ml when the virus was treated with media as a control. These results indicate that NE effectively inactivates RSV at greater than 10 fold lower concentrations than the NE concentrations in the vaccine formulation.

### NE-RSV immunization induces durable RSV-specific antibodies

**Figure 2:**
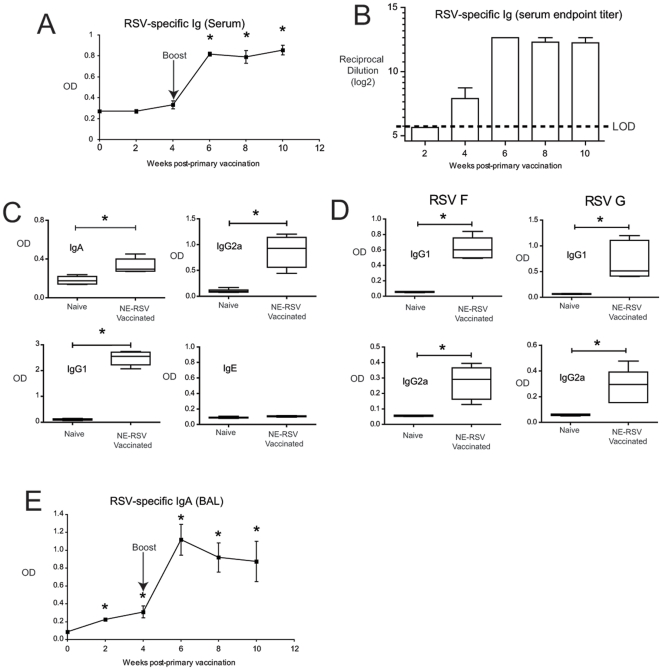
Intranasal vaccination of mice with NE-RSV results in RSV-specific antibody responses. Mice were immunized with NE-RSV containing 10^5^ virus particles at Day 0 and Day 28. In (A), the levels of RSV specific antibodies (IgG+IgM) in serum were determined at weeks 2, 4, 6, 8, and 10 via ELISA using purified RSV protein. In (B), serum samples were serially diluted to obtain endpoints titers. In (C), RSV-specific IgA, IgG2a, IgG1, and IgE were assessed at week 8 via isotype-specific ELISA. In (D), IgG2a and IgG1 antibodies specific for RSV F and G were assessed at week 8 via isotype-specific ELISA using purified RSV F and G glycoproteins. In (E), RSV-specific IgA responses in bronchoalveolar lavage samples (BAL) were assessed using an isotype-specific ELISA. Each time point represents the mean of a minimum of 5 samples +/− SEM, and the experiment was repeated with similar results. * = P<0.01.

We next determined whether NE-RSV vaccination would promote the type of antibody response associated with protection against subsequent RSV infection. To examine this, mice were immunized with two intranasal doses of NE-RSV (5 ul per nare containing ∼10^5^ virus particles), separated by 28 days. Mice were assessed for the presence of RSV-specific serum antibodies immediately prior to the first vaccination, and then every two weeks thereafter. Significant RSV-specific serum responses were observed following the second vaccination with NE-RSV (Fig. 2A)., corresponding to an endpoint titer of 2^14^ (Fig. 2B). No significant decrease in RSV-specific antibodies was observed up to six weeks after the second vaccination (Fig. 2A). The antibody isotypes promoted by the vaccine included IgA, IgG2a, IgG1, but notably, no increase in IgE was observed (Fig. 2C). IgG1 and IgG2a responses were directed at both RSV F and RSV G surface glycoproteins, as assessed via ELISA using recombinant glycoproteins (Fig. 2D). Again, no virus specific IgE antibodies were detected (data not shown). We next determined whether vaccination could promote the induction of RSV-specific antibodies locally in the lungs. Mice were vaccinated twice with NE-RSV, and the bronchoalveloar lavage fluid (BAL) was collected every two weeks post-vaccination. Vaccination did result in significant increases in IgA in the bronchoalveolar lavage (Fig. 2E). Remarkably, a significant increase in RSV-specific IgA in the lungs was detected as early as week 2 post-vaccination.

To identify whether the antibodies generated in our study displayed cross-reactivity, we utilized sera from vaccinated and primary infected animals with direct ELISAs to other A strain viruses. These viruses included A2, Line19, and a new clinical isolate from Drs. Stokes Peebles and Marty Moore's laboratories, 2–20 (unpublished virus stock). All of these viruses demonstrated a significant reactivity with the vaccinated serum (Table 1). Thus, our serum antibody reactivity appears to have the capacity to provide cross-protective antibodies. Future studies will thoroughly evaluate the in vivo capacity of the vaccine to cross protect animals and increase viral clearance.

**Table 1:** Cross-reactivity of IgG1 antibodies induced by NE-RSV vaccination.

	Line 19	Emory 2–20	A2
**Naïve**	0.047±0.002	0.046±0.001	0.050±0.001
**NE-RSV Vaccinated**	1.995±0.112**	2.172±0.114**	2.095±0.098**
**Primary RSV**	0.567±0.243*	0.601±0.284*	0.609±0.245*

Mice were vaccinated with NE-RSV twice and challenged with live RSV Line 19. Serum from vaccinated (NE-RSV vaccinated) and unvaccinated (Primary RSV) were obtained at day 8 post-challenge. Antibody binding to the various RSV strains was determined via isotype-specific ELISA. Values represent the mean OD ± SEM (N = 3–4 animals per group)

* = p, 0.05 versus naive,

** = p, 0.05 versus primary RSV, as determined by ANOVA followed by Dunnett's post test.

### NE-RSV immunization reduces viral load and immunopathology following upper respiratory challenge

**Figure 3:**
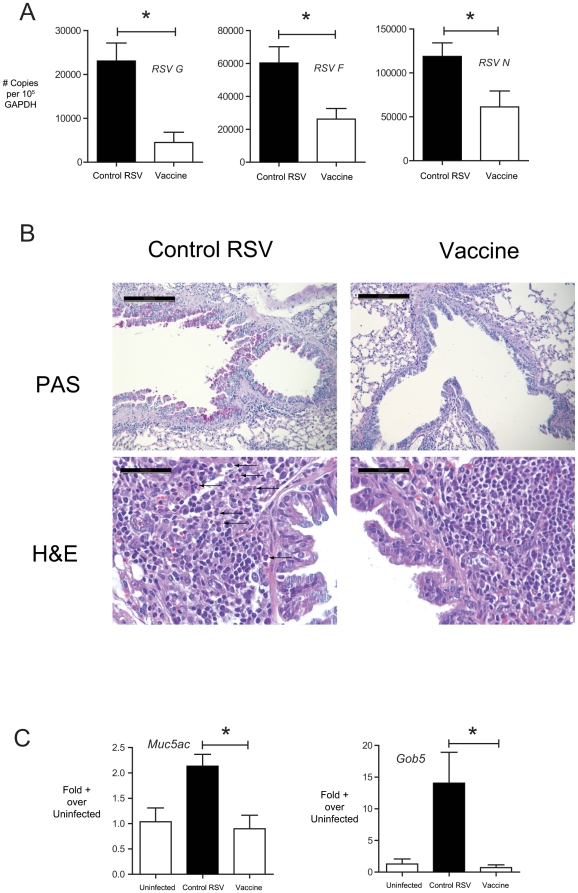
Vaccination of mice with NE-RSV attenuates disease following intranasal challenge with live RSV. Immunized mice were vaccinated intranasally (i.n.) twice at day 0 and day 28 with NE-RSV (10^5^ PFU equivalent). Control and vaccinated mice were challenged at day 56 (i.n.) with 10^5^ PFU live RSV. In (A), the expression of virus transcripts and were determined at day 4 post-infection via QPCR of lung RNA. (B) depicts representative histology (Periodic Acid Schiff's, PAS; Hematoxylin and Eosin, H&E) from control RSV infected and NE-RSV vaccinated mice at day 8 post-infection. In the top two panels, magnification bars are 200 µm in length and 50 µm in the bottom two panels. Arrows indicate airway-associated eosinophils in control RSV infected mice. In (C), the expression of *Muc5ac* and *Gob5* were assessed at day 8 post-infection via QPCR of lung RNA.

**Figure 4:**
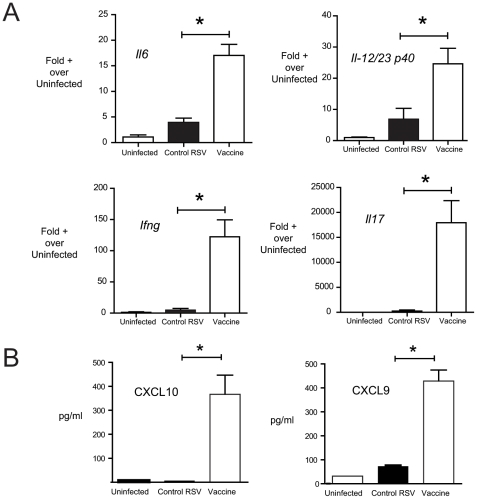
NE-RSV vaccination promotes early Th1/Th17-associated cytokines in the lungs. In (A), the levels of Th1/Th17-associated cytokines were assessed at day 4 post-infection via QPCR of lung RNA. In (B), the levels of CXCL9 and CXCL10 were assessed at day 4 post-infection via Bioplex multiplex assay of lung homogenates. Each column represents 5 mice per group, and the experiment was repeated with similar results. * = p<0.05.

To examine whether vaccination with NE-RSV would affect viral clearance and immunopathology, mice were immunized as above, with two intranasal doses of NE-RSV (at day 0 and day 28), then challenged with live, infectious RSV intranasally (10^5^ PFU) on day 56 post immunization. Intranasal inoculation of mice with Line 19 RSV, leads to an infection that is associated with a moderate form of disease phenotype, including mucus hypersecretion and inflammation. The severity of this phenotype in control and immunized animals was assessed using histologic analysis and QPCR for viral and cytokine gene expression. Compared to control mice, NE-RSV vaccinated mice exhibited enhanced clearance of the virus, as assessed by the levels of RSV transcripts in the lungs at day 4 post challenge (Fig. 3A). At day 8, post-challenge, vaccinated mice exhibited attenuated mucus hypersecretion, as assessed via histologic analysis (Fig. 3B), as well the increased expression of the mucus-associated genes *Muc5ac* and *Gob5* (Fig. 3C). Additionally, no evidence of eosinophilia was observed in mice vaccinated with NE-RSV (5.4%±3.3%) versus controls (6.3%±2.5%) (Fig. 3B). The increased viral clearance was associated with enhanced lung expression of the Th1/Th17 associated cytokines IL-6, IL-12 p40, IFNγ, and IL-17 (Fig. 4A). NE-RSV vaccinated mice also showed enhanced production of chemokines CXCL10 and CXCL9 in the BAL fluid after RSV challenge (Fig. 4B). This was significant because CXCL10 has been previously shown by our laboratory to play a critical role in host defense against RSV via promoting CD8+ T cell responses [21]. These data indicate that NE-RSV vaccination can attenuate RSV-induced upper respiratory disease.

**Figure 5:**
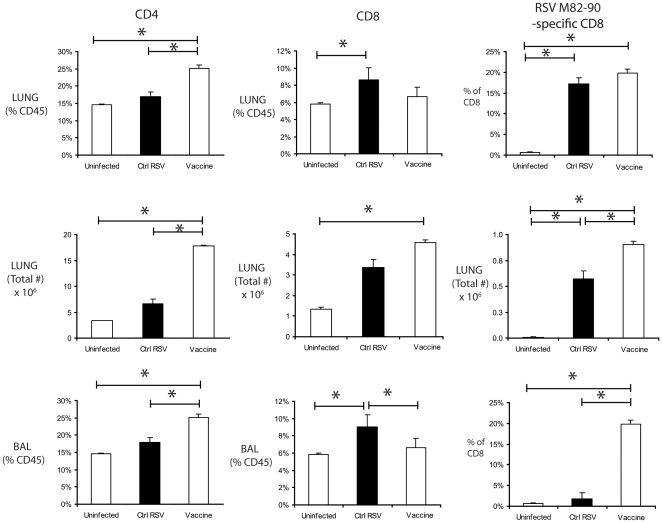
NE-RSV vaccination enhances T cell responses upon live viral challenge. Immunized mice were vaccinated intranasally (i.n.) twice at day 0 and day 28 with NE-RSV (10^5^ PFU equivalent). Control and vaccinated mice were challenged at day 56 (i.n.) with 10^5^ PFU live RSV. The frequency and absolute numbers of total CD4+ T cells, total CD8+ T cells, and RSV M_82–90_ specific CD8+ T cells in the lungs and bronchoalveolar lavage (BAL) were determined at day 8 post-challenge via flow cytometric analysis of BALs and enzymatically digested lungs. * = p<0.05. Each column represents 5 mice per group, and the experiment was repeated with similar results.

We next examined the cellular responses induced by viral challenge in mice following NE-RSV vaccination (Fig. 5). As compared to control RSV-infected mice, NE-RSV vaccinated mice had increased CD4+ T cell responses in the lungs and BAL, and increased RSV M_82–90_ specific CD8+ T cells in the lungs and BAL (Fig. 5). Overall, these data demonstrate that vaccination with NE-RSV promotes T cell-mediated immunity to RSV, including an enhanced CD8+ cytolytic T cell response. This indicates that the cellular immune response to RSV challenge was enhanced for Th1/Tc1 in animals receiving NE-RSV vaccination.

### NE-RSV immunization enhances immunity to lower respiratory RSV challenge

**Figure 6:**
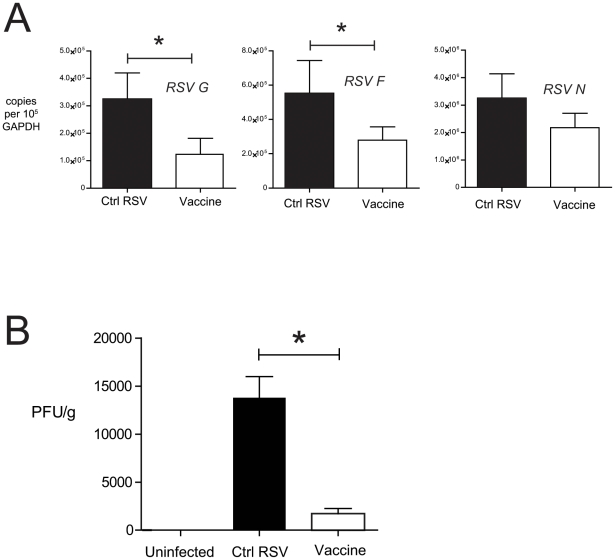
NE-RSV immunization promotes clearance of RSV from the lungs. Mice were immunized (i.n.) as previously described, and challenged oropharyngeally (o.p) with 10^5^ PFU live RSV at day 56. In (A), the expression of viral transcripts in the lungs (day 4 post-infection) was assessed via QPCR. In (B), viral titers were determined at day 4 post-challenge from homogenized lung samples via plaque assay.

Further studies were conducted to determine whether vaccination with NE-RSV would improve viral clearance after a lower respiratory challenge with RSV, which results in a more severe clinical phenotype. Experiments were conducted with an identical immunization and challenge protocol as that employed for intranasal challenge, except that the mice were challenged with RSV intratracheally, rather than intranasally, at Day 56. At day 4 post-challenge, viral load was assessed in the lungs via QPCR and via plaque assay. As assessed via QPCR, a significant decrease in the transcript levels for RSV G and RSV F were detected in the lungs of NE-RSV vaccinated mice (Fig. 6A). A reduction in RSV N was also observed, though this did not reach statistical significance. More compelling, compared to control RSV infected mice, an 88% reduction in plaque forming units (PFU) was found in the lungs of NE-RSV vaccinated mice (Fig. 6B). These data indicate that NE-RSV vaccine dramatically improves viral clearance in the following lower respiratory challenge.

### NE-RSV does not promote airway hyperreactivity, airway eosinophilia, nor the induction of Th2 cytokines

**Figure 7:**
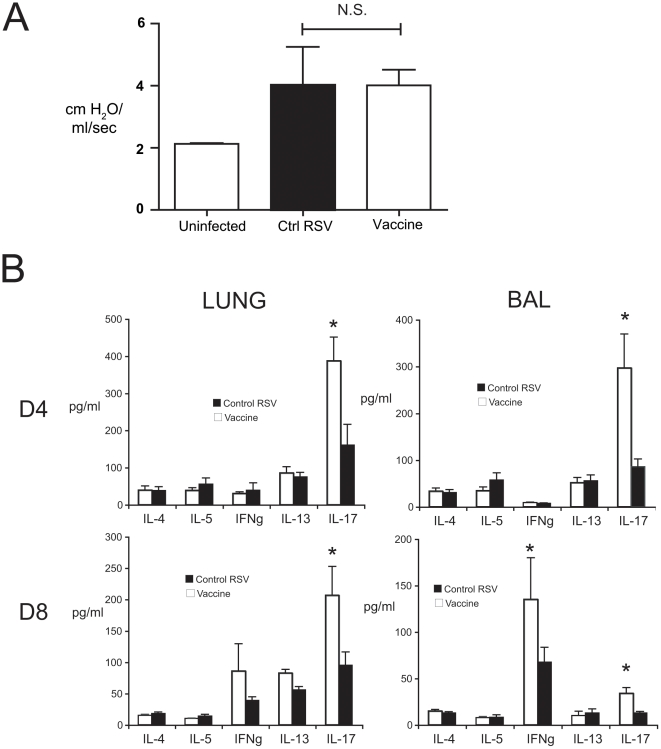
NE-RSV immunization does not promote Th2-mediated immunopotentiation. Mice were vaccinated with NE-RSV as described. Control and vaccinated mice were challenged at day 56. In (A), airway hyperreactivity was assessed at day 8 post-challenge via invasive plethysmography. Columns represent the increase in airway resistance following a single, optimized intravenous dose of methacholine. In (B) cytokine levels in the bronchoalveolar lavage (BAL) and lung homogenate (Lung) were assessed at day 4 and 8 post-challenge. Bars represent the mean of 6–8 mice per group ± SEM. * = p<0.05.

As previously reported, vaccination with FI-RSV promotes the development of airway hyperreactivity (AHR) and eosinophilia upon live viral challenge [22], [23], [24]. Although the cytokine profile and RSV antibody isotype distribution seen in NE-RSV vaccinated mice did not suggest that pathophysiology would be increased by vaccination, we wanted to confirm that vaccination did not promote airway hyper-reactivity, or other evidence of immunopotentiation. Compared to control RSV infected mice, NE-RSV immunized mice exhibited only baseline increases in airway resistance following intravenous methacholine challenge (Fig. 7A). Similarly, no significant eosinophilia was observed in NE-RSV vaccinated mice (Table 2). In contrast, we observed a significant increase in BAL neutrophils after RSV challenge in NE-RSV vaccinated mice with the increased IL-17A, (Table 2). To further examine whether there was any evidence of Th2-mediated immunopotentiation, cytokine profiles were assessed in bronchoalveolar lavage and lung homogenates via multiplex antibody-based assay (Bioplex). NE-RSV vaccination did not result in significantly enhanced Th2 cytokine responses (Fig. 7B), but showed an enhanced IFNγ and IL-17 response (Fig. 7B).

**Table 2:** Neutrophils and eosinophils in the bronchoalveolar lavage (BAL) of uninfected, control RSV infected (Ctrl RSV), and NE-RSV vaccinated (vaccine) mice following oropharyngeal (OP) RSV challenge.

	Neutrophils (%)	Eosinophils (%)
**Uninfected**	1.0±0.0	0.0±0.0
**Ctrl RSV**	5.4±2.4	0.6±0.4
**Vaccine**	15.5±3.0*	5.4±3.3

Mice were vaccinated with NE-RSV and challenged with RSV, as described. Eosinophils and neutrophils were assessed at day 8 post-challenge via differential counts of cytospins. Each represents the mean of 4–6 mice per group +/− SEM.

* = p<0.05 versus control RSV.

**Figure 8:**
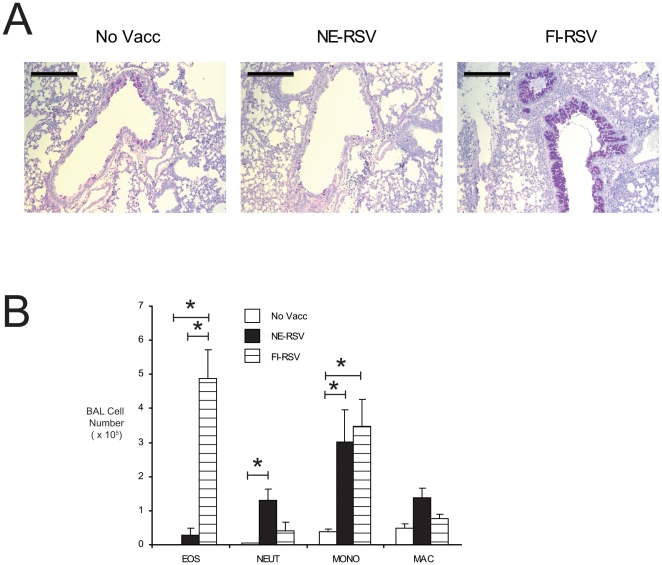
Severe histopathology is associated with formalin-inactivated RSV (FI-RSV) vaccination, but not NE-RSV. Mice were vaccinated with NE-RSV or FI-RSV, as described, and challenged with live RSV. (A) Representative lung histology (Periodic Acid Schiff's, PAS) from unvaccinated control, NE-RSV vaccinated, and FI-RSV vaccinated mice at day 8 post-infection. In (B), the absolute number of eosinophils (Eos), neutrophils (NEUT), small mononuclear cells (MONO), and macrophages (MAC) in the BALs of unvaccinated, NE-RSV vaccinated, and FI-RSV vaccinated mice at day 8 post-infection were determined via hemocytometer and differential staining. * = p<0.05, as assessed by ANOVA followed by Tukey's multiple comparison test. All other pairwise comparisons were not statistically significant.

**Figure 9:**
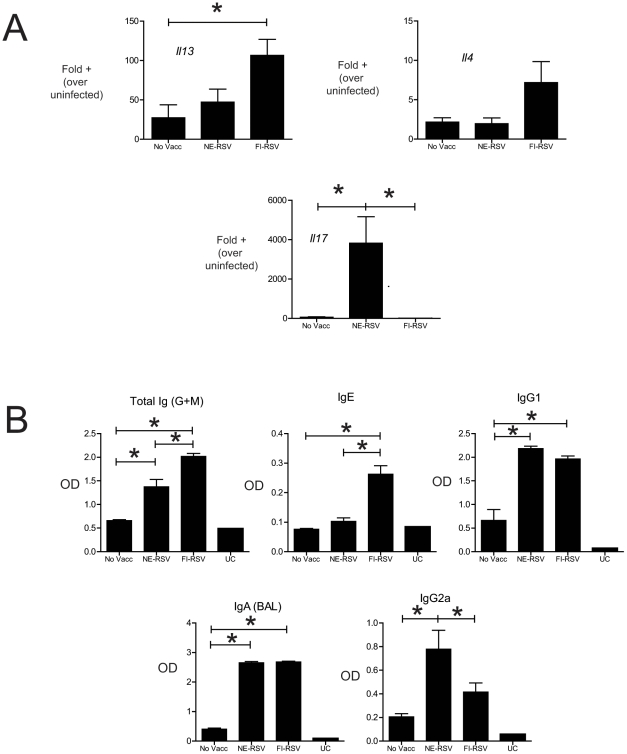
NE-RSV vaccination and FI-RSV vaccination promote distinct cytokine and antibody responses. Mice were vaccinated with NE-RSV or FI-RSV, as described, and challenged with live RSV. In (A), the expression of Th2/Th17 type cytokines was assessed from lung RNA via QPCR. In (B), the levels of RSV specific serum immunoglobulin and BAL IgA were determined by ELISA. Columns represent the mean of five mice per group +/− SEM. * = p<0.05, as assessed by ANOVA followed by Tukey's multiple comparison test. All other pairwise comparisons were not statistically significant.

Since much of the concern regarding RSV vaccines has centered on immunopathology generated by the original formalin inactivated vaccine (FI-RSV), we sought to compare the immune responses generated by NE-RSV versus FI-RSV. In these studies immune responses generated by the two vaccines were directly compared. Mice were vaccinated twice intranasally with NE-RSV, separated by four weeks, as previously described. FI-RSV was administered intramuscularly (IM), six weeks prior to live viral challenge, and mice were assessed at day 8 post-infection. Compared to unvaccinated mice, only FI-RSV vaccinated mice had significantly enhanced mucus hypersecretion (Fig. 8A). Also, neither unvaccinated mice nor NE-RSV vaccinated mice had evidence of airway eosinophilia (Fig. 8B), which is a hallmark of vaccine enhanced disease [25]. In stark contrast to these findings, FI-RSV vaccinated mice had significant airway eosinophilia, with approximately 500,000 total eosinophils in their BAL (Fig. 8B). Consistent with our previous data, NE-RSV vaccination resulted in a more modest increase in neutrophils to the airways (Fig. 8B). Increased mucus and airway eosinophilia in FI-RSV mice were associated with increased expression of IL-13 in the lungs; which was not present in NE-RSV vaccinated mice (Fig. 9A). Consistent with the increase in BAL neutrophils in NE-RSV vaccinated mice, NE-RSV vaccination promoted IL-17 immunity (Fig. 9A). In addition, while vaccination with either NE-RSV or FI-RSV augmented the total RSV-specific antibody response (IgG, IgM, IgG1, and IgA) only FI-RSV vaccinated mice had a significant increase RSV-specific IgE. In contrast, NE-RSV vaccinated mice had increased RSV-specific IgG2a, an isotype associated with Th1 immune responses. Cumulatively, these data demonstrate that NE-RSV promotes clearance of RSV via mechanisms distinct from those induced by FI-RSV.

## Discussion

Five decades of research have documented the mechanisms behind the detrimental immune responses associated with FI-RSV vaccines. The current studies indicate that nanoemulsion-inactivated vaccines, such as NE-RSV, may be a safe alternative to FI-RSV. Given the augmented immune responses observed with the NE-RSV vaccine, this approach could be important for vaccinating immunocompromised patients and infants with underdeveloped immune systems. The vaccine is scalable, and large quantities of virus stocks could be inactivated without the use of toxic agents, such as formaldehyde or beta-propiolactone. Since the vaccine is intrinsically microbicidal, vaccine could potentially be produced and stored without preservatives, such as thimerisol. The intranasal delivery avoids the use of needles, and, while potentially mildly unconfortable, is pain-free. Additionally, the ambient temperature stability of NE-based vaccines makes them attractive candidates for use in developing countries, where maintenance of cold storage is problematic. The functional application of the NE-RSV mucosal vaccine via a non-invasive intranasal route also allows for easy and safe approaches to vaccination, especially in children, and focuses the immune response to the proper location to control viral infections in the respiratory tract.

Our data indicates that NE-RSV vaccine-induced protection from RSV infection is associated with enhanced Th1 and Th17 responses. Though the role of Th1/Tc1 responses to RSV is well-delineated, less is known about the role Th17/Tc17-mediated responses play in RSV host defense [26]. IL-17A, and other members of the IL-17 family have well-described roles in host defense against extracellular bacteria [27], [28]. Conversely, inappropriate activation of the Th17 axis of inflammation contributes to autoimmune disease [29], [30]. The role of IL-17 in pulmonary host defense against viral pathogens remains less well explored and may be dose or pathogen dependent. IL-17 clearly has effects on virus infected cells, leading to enhanced induction of IL-8 [31]. IL-17 producing CD4+ and CD8+ T cells protect mice against viral challenge, however cytotoxicity of virally-infected cells by Tc17 cells may depend on the viral pathogen. Prior work has supported this concept as Tc17 cells are cytolytic against vaccinia but not influenza [32], [33], [34]. Transgenic vaccinia virus expressing IL-23, a Th17 promoting cytokine, is cleared faster than WT virus, in an IL-17-dependent manner, suggesting that IL-23 promotes clearance of vaccinia via IL-17 [35]. Thus, IL17 may be important in clearance of RSV from the lungs of infected individuals and our future experiments will specifically and thoroughly address whether IL-17 has a role in the protective response in this vaccination strategy.

Several mechanisms may play a role in IL-17 mediated responses to RSV. Evidence suggests that there is cross-regulation of Th2 and Th17 responses and therefore IL-17 may serve to antagonize the development of Th2 responses during RSV vaccination [36]. Additionally, IL-17 may play other roles in pulmonary host defense as well, including augmentation of pulmonary antibody responses [37]. The chief mechanism ascribed to IL-17 and Th17 cells in the context of bacterial infection and autoimmune disease is via the recruitment and activation of neutrophils. Neutrophil recruitment, in turn, mediates pathogen clearance or host cell damage. The role of neutrophils in antiviral immunity remains somewhat enigmatic, but neutrophils produce defensins and can participate in complement-mediated lysis of RSV infected cells [38], [39]. Other studies from our laboratories suggest that IL-17 may play a multivariate role in RSV infection. We recently showed that efferent neutralization of IL-17 in TLR7−/− mice (which produce high levels of IL-17 in response to RSV) results in attenuated mucus hypersecretion [40]. Conversely, neutralization of IL-17 in RSV infected mice post-syngeneic bone marrow transplant impairs viral clearance (Lindell et al., Manuscript in Preparation). Though we do not yet know the role of IL-17 in RSV vaccination, IL-17/Th17 differentiation is negatively regulated by IFNα [41], the induction of which may be lower in the context of an inactivated virus vaccine and therefore allow the advantageous effects of IL-17 to prevail.

The coexistence of both IgG1 and IgG2a RSV-specific antibodies observed in the absence of IgE in the NE-RSV vaccinated animals was unanticipated, but both isotypes were similarly induced in the serum of non-vaccinated Balb/C mice undergoing secondary RSV infection (data not shown). These data suggest that the immunoglobulin isotypes induced by NE-RSV vaccination are similar to those induced by live viral infection. Immunoglobulin class switch to IgG2a and IgG2b in mice is promoted by IFNγ, whereas IL-4 promotes IgG1 and IgE [42], [43]; however IL-4 differentially regulates IgG1 and IgE and low levels of IL-4 can promote IgG1 isotype switch, in the absence of the high levels of IL-4 needed to drive IgE production and suppress IgG1 [44]. Comparatively less is known about the role of IL-17 in antibody isotype switching, although one recent study showed that Th17 cells promote Ig class switching *in vitro* and *in vivo* [45]. By using an adoptive transfer system, the latter studies demonstrate that IL-21 (also produced by Th17 cells), rather than IL-17, was critical for the induction of IgG1 [45]. Consistent with a role for IL-21 in promoting IgG1, we observed up-regulation of IL-21 message in NE-RSV vaccinated mice four days post-RSV challenge (624.6±265.4 fold increase, versus a 5.4±3.8 fold increase in unvaccinated controls when comparing both to uninfected mice; p<0.01).

The route of vaccination for NE-RSV (intranasal) may reflect a predominantly mucosal immune response. The use of this particular adjuvant allows the generation of a local, effective immune responses that do not lead to damaging immunopathology after RSV exposure. Recent studies suggest that the addition of TLR agonists as adjuvants can enhance the efficacy of vaccines applied to the mucosal compartment [46], [47], [48]. While it is yet to be determined whether the nanoemulsion provided TLR signals during immunization, further studies with NE-RSV could examine this possibility.

A number of vaccine approaches to RSV have thus far been tested and/or are currently under consideration, and each of these has distinct advantages and disadvantages [49], [50], [51], [52]. These studies indicate that NE-RSV based vaccination promotes both humoral and cellular immune responses to RSV, and effectively improves viral clearance, while not promote detrimental, Th2-mediated immunopathology. This makes a NE-based, inactivated RSV vaccine an attractive approach to provide safe and effective protection against RSV-mediated pulmonary disease.

## Materials and Methods

### Mice

Balb/C mice were purchased from Jackson Laboratories. All animal work was performed in accordance with the University of Michigan Committee on Use and Care of Animals policy or the Institutional Animal Care and Use Committee at Seattle Childrens Research Institute.

### Virus & Vaccine

The nanoemulsion (NE) adjuvant material W_80_5EC used for these studies was produced by NanoBio. NE is an oil-in-water emulsion manufactured from ingredients that are Generally Recognized As Safe (GRAS) with a cationic detergent (cetylpyridinium chloride, CPC) proven safe for human use. The vaccine is administered nasally and it has shown long-term stability at room temperature [11]. Mice were vaccinated and challenged with a subgroup A strain of RSV, referred to as Line 19, originally isolated from a sick infant at the University of Michigan [53]. Line 19 has been demonstrated in animal models to mimic human infection by stimulating mucus production using an inoculum of 1×10^5^ pfu/mouse by intratracheal or oropharyngeal administration [54]. For cross-reactivity assays, RSV A2 was obtained from ATCC and Vanderbilt A200 2–20 was provided by Martin Moore (Emory University). Formalin-inactivated RSV vaccine was prepared following previously published protocols [55], [56]. Briefly, virus stocks were incubated in the presence of 4% neutral buffered formalin for 72 hours at 37 degrees. Virus was pelleted from supernatants via centrifugation for 17 hours at 17,000× g. The pellet was resuspended in media and precipitated with 0.05 M aluminum potassium sulfate, centrifuged at low speed to recover adsorbed virus (1000× g for mins), and resuspended in serum-free media. Mice were vaccinated intramuscularly (i.m.) with 100 ul containing 5×10^5^ PFU equivalent.

### Measurement of airway hyperreactivity

AHR was assessed as previously described [57], [58], [59], [60], [61], [62], [63], [64]. AHR was measured using a Buxco mouse plethysmograph which is specifically designed for the low tidal volumes (Buxco). The mouse to be tested was anesthetized with sodium pentobarbital and intubated via cannulation of the trachea with an 18-gauge metal tube. The mouse was subsequently ventilated with a Harvard pump ventilator (tidal volume = 0.4 ml, frequency = 120 breaths/min, positive end-expiratory pressure 2.5–3.0 cm H2O). The plethysmograph was sealed and readings monitored by computer. As the box is a closed system, a change in lung volume will be represented by a change in box pressure (Pbox) that was measured by a differential transducer. Once baseline levels had stabilized and initial readings were taken, a methacholine challenge was given via tail vein injection. After determining a dose–response curve (0.01–0.5 mg), an optimal dose was chosen, 0.250 mg of methacholine. This dose was used throughout the rest of the experiments in this study. After the methacholine challenge, the response was monitored and the peak airway resistance was recorded as a measure of airway hyperreactivity.

### Quantitative PCR

The smallest lung lobe was removed and homogenized in 1 ml of Trizol reagent (Invitrogen). RNA was isolated as per manufacturer's protocol, and 5 µg was reverse-transcribed to assess gene expression. Detection of cytokine mRNA in lung samples was determined using pre-developed primer/probe sets (Applied Biosystems) and analyzed using an ABI Prism 7500 Sequence Detection System (Applied Biosystems). Transcript levels of *Muc5ac*, *Gob5* were determined using custom primers, as previously described [65]. *Gapdh* was analyzed as an internal control and gene expression was normalized to *Gapdh*. Fold changes in gene expression levels were calculated by comparison to the gene expression in uninfected mice, which were assigned an arbitrary value of 1. RSV transcripts were amplified using SYBR green chemistry, by adapting previously published primer sets to match the sequence of Line 19 [66], [67]:

RSVG sense: 5′-CCAAACAAACCCAATAATGATTT-3′

RSVG antisense:5′-GCCCAGCAGGTTGGATTGT-3′

RSVN sense: 5′-CATCTAGCAAATACACCATCCA- 3′

RSVN antisense: 5′-TTCTGCACATCATAATTAGGAGTATCAA – 3′

RSVF sense: 5′- AATGATATGCCTATAACAAATGATCAGAA-3′

RSVF antisense: 5′- TGGACATGATAGAGTAACTTTGCTGTCT-3′

The levels of RSV transcripts in the lungs were expressed relative to the number of copies of GAPDH.

### Plaque Assays

Lungs of mice were excised, weighed, and homogenized in 1× EMEM (Lonza). Homogenates were clarified by centrifugation (5000× g for 10 mins), serial dilutions were made of the supernatant and added to subconfluent Vero cells. After allowing the virus to adhere for one hour, the supernatant was removed, and replaced with 0.9% methylcellulose/EMEM. Plaques were visualized on day 5 of culture by immunohistochemical techniques using goat anti-RSV as the primary antibody (Millipore), HRP-rabbit anti-goat antibody as the secondary, and 4-chloronapthol (Pierce) as the substrate.

### Serum/BAL ELISA

RSV-specific antibodies were assessed using standard ELISA techniques. Briefly, high binding 96-well plates were coated with purified, inactivated RSV (US Biological). After blocking, serial dilutions of serum or BAL were added to the plates and incubated overnight at 4 degrees. Bound antibodies were detected using an HRP-conjugated goat anti-mouse IgG/IgM/IgA cross-reactive antibody(AbD Serotec), or using isotype specific detection antibodies (AbD Serotec), and developed with TMB substrate (KPL). RSV F and G specific ELISAs were performed similarly, except that plates were coated with purified recombinant RSV F or RSV G glycoproteins (1.3 ug/ml) expressed in Baculovirus (Sino Biological). Endpoint titers were determined via the method of Frey, Di Canzio, and Zurakowski, in which the OD cutoff is a statistically defined increase over the OD of samples from naive animals at the same dilution [68]. Cross-reactivity ELISAs were performed by coating plates with clarified viral supernatant from RSV infected Hep2 cells.

### Lung and Lymph Node Leukocyte Isolation

Lung leukocytes were isolated from enzyme dispersed lung tissue. Right lungs from each mouse were excised, washed in PBS, minced and digested enzymatically for 45 minutes in 15 ml/lung of digestion buffer (RPMI, 5% FCS, 1 mg/ml collagenase (Roche Applied Science), and 30 ug/ml DNase (Sigma-Aldrich). Lung-associated lymph nodes (LALN) were dispersed similar to lungs, except that only 5 mls of digestion buffer was used. Following erythrocyte lysis using ammonium chloride (NH_4_Cl) buffer, cells were washed, and resuspended in media (RPMI, 5% FCS). Total lung leukocyte numbers were assessed in the presence of trypan blue using a hemocytometer; viability was >85%.

### Lymph node restimulation

Lung associated lymph node (LALN) cell suspensions were plated in duplicate at 1×10^6^ cells per well followed by restimulation with either media or RSV (MOI∼0.5). Cells were incubated at 37°C for 24 hours and supernatants collected for analysis on the BioRad Bioplex 200 system according to the manufacturer's protocol. Kits (BioRad) containing antibody beads to Th cytokines (IL-17, IFNγ, IL-4, IL-5, IL-13) were used to assay for cytokine production in each of the samples.

### Lung Protein Analysis

The levels of chemokines and cytokines in lung homogenates were assessed using multiplex bead-based arrays (Invitrogen), and analysed on the Bioplex 200 system (BioRad).

### Flow cytometric analysis

All antibodies for flow cytometry (BD Pharmingen, Biolegend, or eBioscience) were used to stain intact cells per manufacturer's instructions, and analysed on an LSR II cytometer (BD Biosciences). RSV M_82–90_ specific CD8+ T cells were stained with H-2Kd-restricted SYIGSINNI Pro5® MHC Class I Pentamer reagent (Proimmune). Isotype control antibodies were used to demonstrate specificity of all stains and to establish the criteria for designated flow cytometry populations.

### Histology

Right lobes of the lungs were isolated and immediately fixed in 10% neutral buffered formalin. Lung samples were subsequently processed, embedded in paraffin, sectioned, and placed on L-lysine-coated slides, and stained using standard histological techniques using Hemotoxylin and Eosin (H&E) and Periodic-acid Schiff (PAS). PAS staining was done to identify mucus and mucus-producing cells.

### Statistics

Data was analyzed using Prism GraphPad software. Unless otherwise specified, data shown is representative of two or more experiments. Statistical significance in all experiments was determined by One-way ANOVA, followed by a Newman-Keuls post test. Significant differences were regarded as p<0.05. Statistical comparisions of QPCR data were determined from normalized Ct values, before conversion to fold-increases.
